# Association of hemoglobin levels at admission with postoperative pneumonia in elderly patients with hip fracture: A retrospective cohort study

**DOI:** 10.1097/MD.0000000000034270

**Published:** 2023-07-28

**Authors:** Daxue Zhang, Ning Zhang, Lixin Sun, Yu Zhang, Shiwei Yang

**Affiliations:** a School of Nursing, Anhui Medical University, Hefei, China; b Teaching Office, Shenzhen Second People’s Hospital, Shenzhen, China; c First Affiliated Hospital of Shenzhen University, Shenzhen, China; d Department of Orthopedics, Zhejiang Hospital, Hangzhou, China.

**Keywords:** anemia, hemoglobin, hip fracture, postoperative pneumonia

## Abstract

Previous studies have suggested a correlation between low preoperative hemoglobin (Hb) levels and postoperative pneumonia (POP) in elderly patients with hip fractures. However, the exact inflection point of Hb level that increases the risk of POP remains unclear. This study aimed to investigate the quantitative relationship between preoperative Hb levels and the incidence of POP in this patient population. This retrospective study included 1417 elderly patients with hip fractures who were admitted to the Department of Orthopedics at Shenzhen Second People’s Hospital between January 2012 and December 2021. Demographic and clinical data, including laboratory test results, were analyzed and compared to explore the relationship between Hb levels at admission and the incidence of POP in this patient population. This study included 1417 elderly patients with hip fractures, comprising 382 males and 1035 females, with a mean age of 77.57 ± 8.73 years. The incidence of POP was 6.21% (88/1417) in this patient population. After adjusting for confounding factors in model II, the regression equation showed that the incidence of POP decreased by 2% with each 1 g/L increment in Hb levels (OR: 0.98, 95% CI: 0.97–1.00; *P* = .0211). Additionally, a two-piecewise regression model was used to explore the relationship between Hb levels and POP incidence, after adjusting for confounding factors. Threshold effect analysis showed that the inflection point was 83.5 g/L. On the left side of the inflection point, Hb levels were negatively correlated with the incidence of POP (OR: 0.91, 95% CI: 0.86–0.97, *P* = .0030). There was a nonlinear relationship between preoperative Hb level and POP in elderly patients with hip fractures. When Hb levels were lower than 83.5 g/L, preoperative Hb levels were negatively correlated with POP.

## 1. Introduction

Postoperative pneumonia (POP) is a common complication in elderly patients with hip fractures, with reported incidence rates ranging from 5.1% to 14.9%.^[1–3]^ The increasing number of hip fracture surgeries performed globally over the past few decades has made POP a significant concern for the healthcare system. Epidemiological evidence indicates that POP is associated with a significant increase in 30-day mortality, hospital stay duration, and readmission.^[4–6]^ These findings underscore the importance of identifying the potential risk factors for POP in elderly patients with hip fractures to reduce the burden of this complication on the healthcare system and improve patient outcomes.

Anemia is a common condition in patients with hip fractures due to advanced age, underlying diseases, malnutrition, and trauma. It is also associated with adverse postoperative outcomes. According to the findings of the study,^[7]^ patients with hip fractures who also had anemia experienced prolonged hospitalization, increased prevalence of POP, and higher hospitalization expenses. Previous studies have reported that preoperative anemia is present in 12.8% to 24.3% of patients undergoing hip and knee arthroplasty.^[8,9]^ Domestic studies have shown that the incidence of preoperative anemia in patients undergoing hip replacement was 25.6% in males and 32.8% in females, which has been associated with increased postoperative mortality, delayed recovery, and decreased quality of life.^[10]^

Several recent studies have suggested that low hemoglobin (Hb) levels are an independent risk factor for POP in elderly patients with hip fractures.^[4,11]^ However, the literature remains controversial regarding this finding,^[12]^ and a definitive Hb cutoff value for the risk of POP in elderly patients with hip fractures has yet to be established. Therefore, this retrospective study aimed to analyze the clinical data of elderly patients with hip fractures in our hospital to explore the quantitative correlation between Hb levels at admission and POP, with the goal of providing a reference Hb level to guide preoperative blood transfusions in elderly patients with anemia.

## 2. Methods

### 2.1. Population

This study was a retrospective cohort analysis of 1853 elderly patients who were admitted to Shenzhen Second People’s Hospital between January 2012 and December 2021. The inclusion criteria for this retrospective cohort study were as follows: X-ray-confirmed hip fracture (femoral neck or intertrochanteric fracture) and age > 60 years. The exclusion criteria were as follows: old fracture, defined as the time from fracture to admission exceeding 3 weeks; pathological fracture; periprosthetic femoral fracture; multiple or open fractures; preoperative diagnosis of pneumonia; complications with hematologic and immune system diseases, such as leukemia, rheumatoid arthritis, and systemic lupus erythematosus; no surgery; and missing data. After exclusion screening, 1417 patients were included in the study. The mean age of the patients included in the study was 77.55 ± 8.75 years, with 389 males (26.94%) and 1055 females (73.06%). This study was conducted in accordance with the principles outlined in the *Declaration of Helsinki* and was approved by the *Clinical Research Ethics Committee of Shenzhen Second People’s Hospital* (No. 20210620213357012-FS01). The study protocol was registered at ClinicalTrials.gov (registration number: ChiCTR2100047560). Because patient information was collected retrospectively and anonymized, the requirement for informed consent was waived.

### 2.2. Definition of pneumonia

The diagnostic criteria for pneumonia were based on the guidelines for the diagnosis and treatment of adult hospital-acquired and ventilator-associated pneumonia in China (2018 edition).^[13]^ These criteria included a clinical diagnosis of POP based on the presence of a new or progressive infiltrative, solid, or ground-glass shadow on chest X-ray or computed tomography, along with 2 or more of the following 3 clinical signs: fever with a temperature > 38.0°C, purulent airway discharge, and peripheral blood leukocyte count > 10 × 10^9/L or < 4 × 10^9/L. The patient did not have any other pulmonary diseases, such as tuberculosis, lung cancer, or pulmonary embolism, and was diagnosed with pneumonia. The outcome measure of this study was POP, which was observed during the hospitalization period starting 24 hours after the operation.

### 2.3. Exposed variable

The target exposure variable in this study was the Hb level at admission, which was recorded as a continuous variable. Fasting blood samples were collected within 24 hours of admission, with a fasting period of > 8 hours. Hb levels were automatically determined using a blood cell analyzer and colorimetry.

### 2.4. Covariate variables

Clinical data from patients were collected using electronic and medical records from the hospital. The following data were collected: age, sex, body mass index, fracture classification, number of comorbidities, time from fracture to surgery, smoking status, medical history (including hypertension, coronary heart disease, stroke, and diabetes mellitus), American Society of Anesthesiologists (ASA) classification, operation method, anesthesia method, operation duration, intraoperative blood loss, intensive care unit (ICU) admission, in-hospital death, length of hospital stay, white blood cell (WBC) count, lymphocyte count, neutrophil count, platelet count, red blood cell distribution width (RDW), albumin, globulin, serum creatinine, and blood urea nitrogen (BUN). Fasting blood tests (>8 hours) were performed within 24 hours of admission for all laboratory indicators.

### 2.5. Statistical analysis

Continuous variables are presented as mean ± standard deviation (normal distribution) or median (quartile) (skewed distribution), while categorical variables are expressed as frequencies or percentages. One-way Analysis of Variance (normal distribution), Kruskal–Wallis *H* (skewed distribution) test, and chi-square tests (categorical variables) were used to determine statistical differences between the means and proportions of the groups. A univariate linear regression model was used to evaluate the association between Hb levels and POP using both non-adjusted and multivariate-adjusted models. According to the recommendations of the STROBE statement, the results of the unadjusted, minimally adjusted, and fully adjusted analyses were presented simultaneously. Covariances were adjusted based on the following principle: when added to the model, the matched odds ratio should change by at least 10%.^[14]^ A generalized additive model was applied to identify nonlinear relationships. If a nonlinear correlation was observed, a two-piecewise linear regression model was used to calculate the threshold effect of Hb level on pneumonia using a smoothing plot. When the ratio between POP and Hb clearly appeared in the smoothed curve, a recursive method was applied to automatically calculate the inflection point at which the maximum likelihood model was used.^[15]^ The modification and interaction of the subgroups were examined using the likelihood ratio test. All analyses were performed using statistical software packages R (http://www.R-project.org, The R Foundation) and EmpowerStats (http://www.empowerstats.com, X&Y Solutions, Inc., Boston, MA). *P* < .05 (two-sided) were considered statistically significant.

## 3. Results

### 3.1. Description of the patient screening process

**Figure 1.**
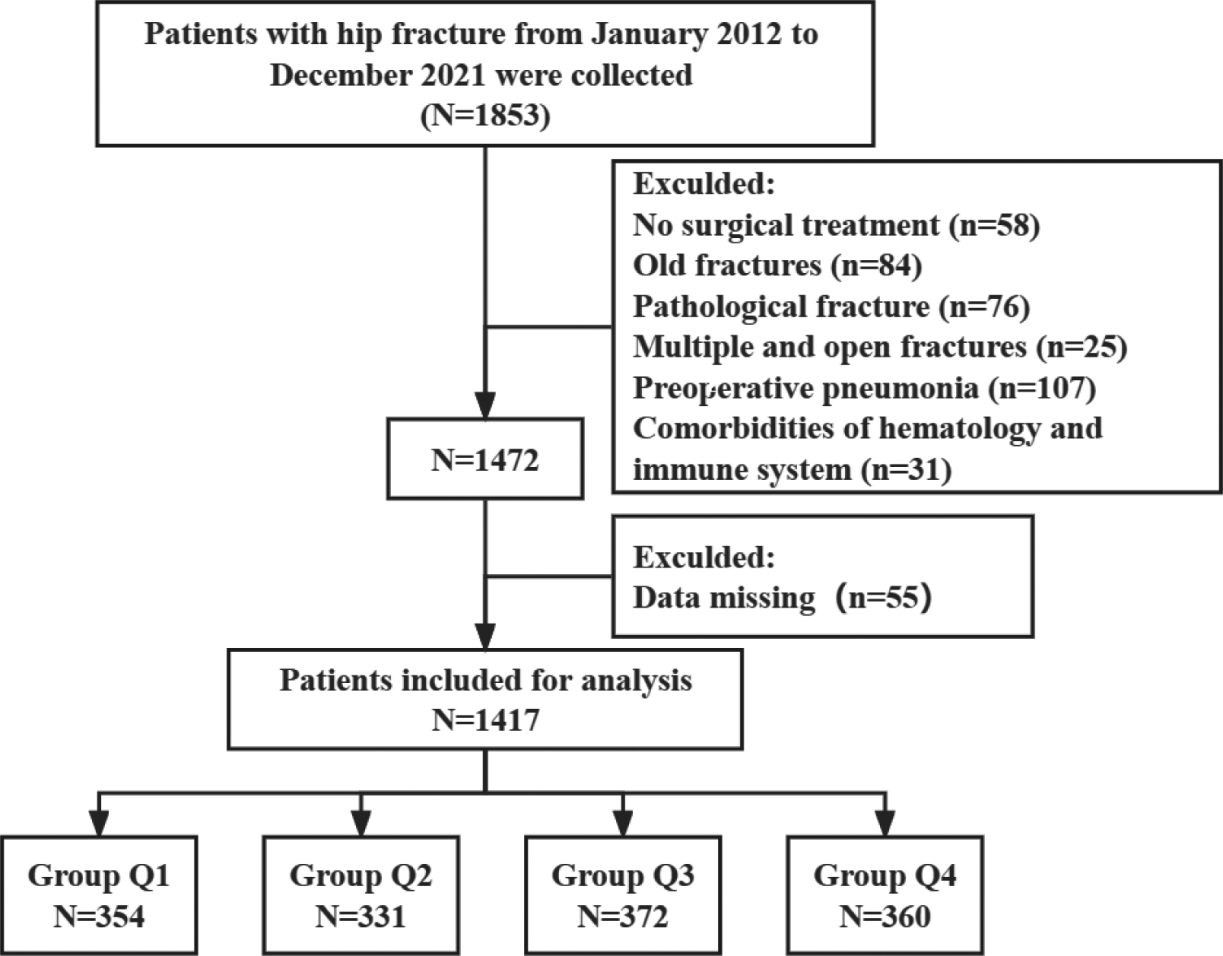
Flow chart of subject selection.

A total of 1853 elderly patients with hip fractures admitted to Shenzhen Second People’s Hospital between January 2012 and December 2021 were included in this study. 58 patients without surgical treatment, 84 with old fractures, 76 with pathological fractures, 25 with multiple open fractures, 107 with preoperative pneumonia, 31 with hematological and immune system diseases, and 55 with incomplete data were excluded. Thus, 1417 patients were included in the final analysis. See the flowchart for details (Fig. 1).

### 3.2. Baseline characteristics of participants

The distributions of the baseline characteristics are presented in Table 1. To group Hb in quartiles, Hb data were ordered from smallest to largest, and the ordered distribution was divided into 4 parts of roughly equal size (Q1–Q4). After grouping, the distribution trends of each variable among different groups were observed. The mean age of 1417 patients was 77.57 ± 8.73 years, and the incidence of POP was 6.21% (88/1417). Compared with the Q4 group, the Q1, Q2, and Q3 groups had higher mean ages, higher incidences of POP, and higher proportions of female patients and femoral neck fractures (all *P* < .05). In contrast, the body mass index, lymphocyte count, neutrophil count, white blood cell count, and albumin level in the Q1, Q2, and Q3 groups were lower than those in the Q4 group (all *P* < .05). No significant differences were observed in the length of hospital stay, operation duration, monocyte count, platelet count, globulin level, in-hospital death, ICU admission, smoking status, hypertension, stroke, diabetes mellitus, or anesthesia method among the 4 Hb groups (all *P* > .05).

**Table 1:** Baseline characteristics of participants 1417.

Hb (g/L)	Q1 91.93 (10.92)	Q2 111.34 (3.79)	Q3 123.24 (3.36)	Q4 138.94 (8.44)	*P* value
Number (n)	354	331	372	360	
Age (yr)	81.07 (7.97)	78.04 (8.70)	76.98 (8.56)	74.29 (8.34)	<.001
Sex (n, %)					
Male	94 (26.55%)	58 (17.52%)	95 (25.54%)	135 (37.50%)	<.001
Female	260 (73.45%)	273 (82.48%)	277 (74.46%)	225 (62.50%)	
BMI (kg/m^2^)	20.98 (3.14)	21.55 (3.03)	22.30 (3.35)	23.26 (3.01)	<.001
Classification of fracture (n, %)					
Femoral neck fracture	127 (35.88%)	199 (60.12%)	281 (75.54%)	296 (82.22%)	<.001
Intertrochanteric fracture	227 (64.12%)	132 (39.88%)	91 (24.46%)	64 (17.78%)	
Time from fracture to surgery (h)	92.00 (58.50–149.75)	75.00 (48.00–136.00)	71.00 (48.00–120.00)	76.00 (50.75–136.25)	.002
Number of comorbidities (n, %)					
≤3	314 (88.70%)	311 (93.96%)	350 (94.09%)	332 (92.22%)	.025
>3	40 (11.30%)	20 (6.04%)	22 (5.91%)	28 (7.78%)	
Smoking status (n, %)					
No	337 (95.20%)	320 (96.68%)	354 (95.16%)	341 (94.72%)	.639
Yes	17 (4.80%)	11 (3.32%)	18 (4.84%)	19 (5.28%)	
Hypertension (n, %)					
No	176 (49.72%)	189 (57.10%)	190 (51.08%)	179 (49.72%)	.169
Yes	178 (50.28%)	142 (42.90%)	182 (48.92%)	181 (50.28%)	
CHD (n, %)					
No	297 (83.90%)	290 (87.61%)	322 (86.56%)	323 (89.72%)	.137
Yes	57 (16.10%)	41 (12.39%)	50 (13.44%)	37 (10.28%)	
Stroke (n, %)					
No	286 (80.79%)	281 (84.89%)	310 (83.33%)	288 (80.00%)	.303
Yes	68 (19.21%)	50 (15.11%)	62 (16.67%)	72 (20.00%)	
DM (n, %)					
No	262 (74.01%)	265 (80.06%)	300 (80.65%)	267 (74.17%)	.047
Yes	92 (25.99%)	66 (19.94%)	72 (19.35%)	93 (25.83%)	
WBC count (×10^9/L)	8.80 (3.31)	8.95 (2.90)	9.35 (2.88)	9.78 (2.96)	<.001
Lymphocyte count (×10^9/L)	1.19 (0.59)	1.31 (0.71)	1.32 (0.55)	1.34 (0.52)	.002
Neutrophil count (×10^9/L)	7.14 (4.71)	7.01 (2.73)	7.27 (2.84)	7.71 (2.95)	.037
Monocyte count (×10^9/L)	0.60 (0.45)	0.58 (0.26)	0.60 (0.25)	0.59 (0.24)	.721
Platelet count (×10^9/L)	207.95 (80.88)	206.67 (78.53)	208.03 (75.09)	204.30 (66.81)	.902
RDW (%)	14.19 (2.01)	13.43 (1.32)	13.20 (1.76)	12.97 (0.87)	<.001
Scr (µmol/L)	73.00 (58.25–97.00)	63.80 (52.40–78.25)	62.15 (51.08–75.00)	63.40 (53.00–77.00)	<.001
BUN (mmol/L)	6.20 (4.20–8.20)	4.90 (3.18–6.62)	4.80 (2.96–6.50)	4.70 (2.88–6.10)	<.001
Albumin (g/L)	35.25 (5.08)	38.04 (3.59)	38.81 (3.42)	40.07 (4.08)	<.001
Globulin (g/L)	27.78 (5.32)	28.78 (5.31)	28.29 (4.41)	29.04 (11.64)	.102
ASA classification (n, %)					
≤2	106 (29.94%)	158 (47.73%)	176 (47.31%)	173 (48.06%)	<.001
≥3	248 (70.06%)	173 (52.27%)	196 (52.69%)	187 (51.94%)	
Operation method (n, %)					
Internal fixation	202 (57.06%)	147 (44.41%)	127 (34.14%)	110 (30.56%)	<.001
Hip replacement	152 (42.94%)	184 (55.59%)	245 (65.86%)	250 (69.44%)	
Anesthesia method (n, %)					
Non general anesthesia	260 (73.45%)	251 (75.83%)	292 (78.49%)	276 (76.67%)	.454
General anesthesia	94 (26.55%)	80 (24.17%)	80 (21.51%)	84 (23.33%)	
Operation duration (min)	82.53 (38.20)	87.84 (38.90)	82.61 (31.63)	87.03 (34.09)	.086
Intraoperative blood loss (mL)	200.00 (100.00-300.00)	200.00 (100.00-300.00)	200.00 (100.00-300.00)	200.00 (100.00-400.00)	<.001
ICU admission (n, %)					
No	341 (96.33%)	326 (98.49%)	364 (97.85%)	355 (98.61%)	.143
Yes	13 (3.67%)	5 (1.51%)	8 (2.15%)	5 (1.39%)	
In-hospital death (n, %)					
No	353 (99.72%)	330 (99.70%)	370 (99.46%)	360 (100.00%)	.597
Yes	1 (0.28%)	1 (0.30%)	2 (0.54%)	0 (0.00%)	
Length of hospital stay (d)	10.50 (8.00–16.00)	10.00 (8.00–15.00)	10.00 (8.00–16.00)	10.00 (7.00–15.00)	.303
POP (n, %)					
No	320 (90.40%)	312 (94.26%)	353 (94.89%)	344 (95.56%)	.020
Yes	34 (9.60%)	19 (5.74%)	19 (5.11%)	16 (4.44%)	

ASA = American Society of Anesthesiologists, BMI = body mass index, BUN = blood urea nitrogen, CHD = coronary heart disease, DM = diabetes mellitus, Hb = hemoglobin, ICU = intensive care unit, POP = postoperative pneumonia, RDW = red blood cell distribution width, Scr = serum creatinine, WBC = white blood cell.

### 3.3. Univariate analysis

Univariate analysis revealed that age, sex, number of comorbidities, coronary heart disease, stroke, Hb, WBC count, neutrophil count, RDW, BUN, albumin, ASA classification, intraoperative blood loss, ICU admission, and length of hospital stay were correlated with the incidence of POP (all *P* < .05) (Table 2).

**Table 2:** Univariate analysis of postoperative pneumonia.

	Statistics	95%CI	*P* value
Age (yr)	77.57 ± 8.73	1.07 (1.05, 1.10)	<.0001
Sex (n, %)			
Male	382 (26.96%)	1.0	
Female	1035 (73.04%)	0.63 (0.40, 0.98)	.0416
BMI (kg/m^2^)	22.04 ± 3.25	0.97 (0.91, 1.04)	.4018
Classification of fracture (n, %)			
Femoral neck fracture	903 (63.73%)	1.0	
Intertrochanteric fracture	514 (36.27%)	1.23 (0.79, 1.91)	.3511
Time from fracture to surgery (h)	109.05 ± 89.75	1.00 (1.00, 1.00)	.0840
Number of comorbidities (n, %)			
≤3	1037 (92.24%)	1.0	
>3	110 (7.76%)	2.43 (1.32, 4.46)	.00042
Smoking status (n, %)			
No	1352 (95.41%)	1.0	
Yes	65 (4.59%)	1.89 (0.84, 4.28)	.1250
Hypertension (n, %)			
No	734 (51.80%)	1.0	
Yes	683 (48.20%)	1.19 (0.77, 1.83)	.4303
CHD (n, %)			
No	1232 (86.94%)	1.0	
Yes	185 (13.06%)	1.93 (1.13, 3.29)	.0157
Stroke (n, %)			
No	1165 (82.22%)	1.0	
Yes	252 (17.78%)	2.44 (1.53, 3.89)	.0002
DM (n, %)			
No	1094 (77.21%)	1.0	
Yes	323 (22.79%)	1.29 (0.79, 2.10)	.3023
Hb (g/L)	116.63 ± 18.74	0.98 (0.97, 0.99)	.0002
WBC count (×10^9/L)	9.23 ± 3.04	1.10 (1.03, 1.17)	.0033
Lymphocyte count (×10^9/L)	1.29 ± 0.59	0.73 (0.47, 1.13)	.1555
Neutrophil count (×10^9/L)	7.29 ± 3.41	1.06 (1.01, 1.11)	.0187
Monocyte count (×10^9/L)	0.59 ± 0.31	1.10 (0.58, 2.08)	.7650
Platelet count (×10^9/L)	206.74 ± 75.36	1.00 (1.00, 1.00)	.6986
RDW (%)	13.45 ± 1.62	1.16 (1.05, 1.28)	.0024
Scr (µmol/L)	81.15 ± 81.77	1.00 (1.00, 1.00)	.0696
BUN (mmol/L)	5.62 ± 3.77	1.05 (1.01, 1.10)	.0131
Albumin (g/L)	38.06 ± 4.46	0.92 (0.88, 0.96)	.0001
Globulin (g/L)	28.46 ± 7.30	1.02 (1.00, 1.04)	.0506
ASA classification (n, %)			
≤2	613 (43.26%)	1.0	
≥3	804 (56.74%)	2.00 (1.24, 3.22)	.0043
Operation method (n, %)			
Internal fixation	586 (41.35%)	1.0	
Hip replacement	831 (58.65%)	1.39 (0.88, 2.19)	.1546
Anesthesia method (n, %)			
Non general anesthesia	1079 (76.15%)	1.0	
General anesthesia	338 (23.85%)	1.00 (0.60, 1.66)	.9981
Operation duration (min)	84.93 ± 35.77	1.00 (0.99, 1.00)	.1938
Intraoperative blood loss (mL)	244.84 ± 191.79	1.00 (1.00, 1.00)	.0197
ICU admission (n, %)			
No	1386 (97.81%)	1.0	
Yes	31 (2.19%)	14.60 (6.93, 30.76)	<.0001
In-hospital death (n, %)			
No	1413 (99.72%)	1.0	
Yes	4 (0.28%)	5.08 (0.52, 49.35)	.1611
Length of hospital stay (d)	12.07 ± 6.89	1.10 (1.07, 1.12)	<.0001

ASA = American Society of Anesthesiologists, BMI = body mass index, BUN = blood urea nitrogen, CHD = coronary heart disease, DM = diabetes mellitus, Hb = hemoglobin, ICU = intensive care unit, RDW = red blood cell distribution width, Scr = serum creatinine, WBC = white blood cell.

### 3.4. The results of relationship between Hb and POP

Three models were used to evaluate the linear association between Hb levels and POP (Table 3). In the crude model, the incidence of POP decreased by 2% with each 1 g/L increment in Hb levels (OR: 0.98, 95% CI: 0.97–0.99, *P* = .0002). In adjusted models I and II, an association was also identified (OR: 0.99, 95% CI: 0.97–1.00, *P* = .0146) and (OR: 0.98, 95% CI: 0.97–1.00, *P* = .0211), respectively. To perform a sensitivity analysis, we used Hb as a categorical variable (quartile) and observed the same trend in the non-adjusted model (*P* = .0051).

**Table 3:** Relationship between Hb and POP in different models.

Variable	Non-adjusted M, *P* value	Adjusted MI, *P* value	Adjusted MII, *P* value
Hb	0.98 (0.97, 0.99) .0002	0.99 (0.97, 1.00) .0146	0.98 (0.97, 1.00) .0211
Hb quartile			
Q1	1.0	1.0	1.0
Q2	0.57 (0.32, 1.03) .0612	0.70 (0.39, 1.27) .2469	0.81 (0.42, 1.57) .5398
Q3	0.51 (0.28, 0.91) .0219	0.63 (0.35, 1.15) .1322	0.60 (0.27, 1.23) .1629
Q4	0.44 (0.24, 0.81) .0083	0.62 (0.33, 1.18) .1487	0.63 (0.29, 1.39) .2515
*P* for trends	.0051	.1017	.1751

Non-adjusted M (Non-adjusted model): No adjustment.

Adjusted MI (adjusted model I): adjustment for age, sex.

Adjusted MII (adjusted model II): adjustment for age, sex, classification of fracture, number of comorbidities, smoking status, CHD, stroke, WBC, neutrophil, monocyte, RDW, Scr, BUN, albumin, ASA classification, intraoperative blood loss and ICU admission.

ASA = American Society of Anesthesiologists, BUN = blood urea nitrogen, CHD = coronary heart disease, Hb = hemoglobin, ICU = intensive care unit, POP = postoperative pneumonia, RDW = red blood cell distribution width, Scr = serum creatinine, WBC = white blood cell.

### 3.5. Results of nonlinear association between Hb and POP

**Figure 2.**
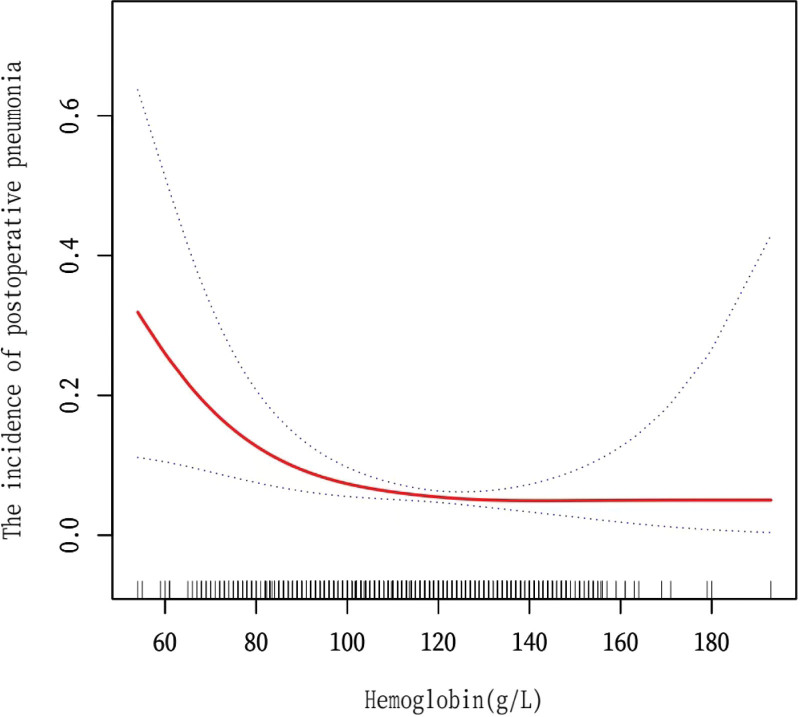
The correlation between Hb and POP in patients with hip fractures. The data were adjusted for age, sex, classification of fracture, number of comorbidities, smoking status, CHD, stroke, WBC, neutrophil, monocyte, RDW, Scr, BUN, albumin, ASA classification, intraoperative blood loss, and ICU admission. ASA = American Society of Anesthesiologists, BUN = blood urea nitrogen, CHD = coronary heart disease, Hb = hemoglobin, ICU = intensive care unit, POP = postoperative pneumonia, RDW = red blood cell distribution width, Scr = serum creatinine, WBC = white blood cell.

Smoothing curve fitting and generalized additive models revealed a nonlinear association between Hb level and POP. Our findings indicated a threshold effect for the association between Hb levels and the incidence of POP after adjusting for age, sex, classification of fracture, number of comorbidities, smoking status, coronary heart disease, stroke, WBC count, neutrophil count, monocyte count, RDW, serum creatinine, BUN, albumin, ASA classification, intraoperative blood loss, and ICU admission (Fig. 2). Using a two-piecewise linear model and recursive algorithm, we identified an inflection point for Hb level of 83.5 g/L. To the left of the inflection point, for every 1 g/L increase in Hb, the risk of POP decreased by 9% (OR: 0.91, 95% CI: 0.86–0.97; *P* = .0030). To the right of the inflection point, the incidence of POP did not change significantly with an increase in the Hb level (*P* = .3399) (Table 4).

**Table 4:** Nonlinearity explanation on Hb and POP using the two-phase linear model.

	Incidence of POP OR (95%CI) *P* value
Model I	
Linear effect	0.98 (0.97, 1.00) .0211
Model II	
Inflection point (Hb)	83.5
<Inflection point	0.91 (0.86, 0.97) .0030
>Inflection point	0.99 (0.98, 1.01) .3399
*P* for log likely ratio test	.019

The data were adjusted for age, sex, classification of fracture, number of comorbidities, smoking status, CHD, stroke, WBC, neutrophil, monocyte, RDW, Scr, BUN, albumin, ASA classification, intraoperative blood loss, and ICU admission.

ASA = American Society of Anesthesiologists, BUN = blood urea nitrogen, CHD = coronary heart disease, Hb = hemoglobin, ICU = intensive care unit, POP = postoperative pneumonia, RDW = red blood cell distribution width, Scr = serum creatinine, WBC = white blood cell.

### 3.6. The results of subgroup analyses

**Figure 3.**
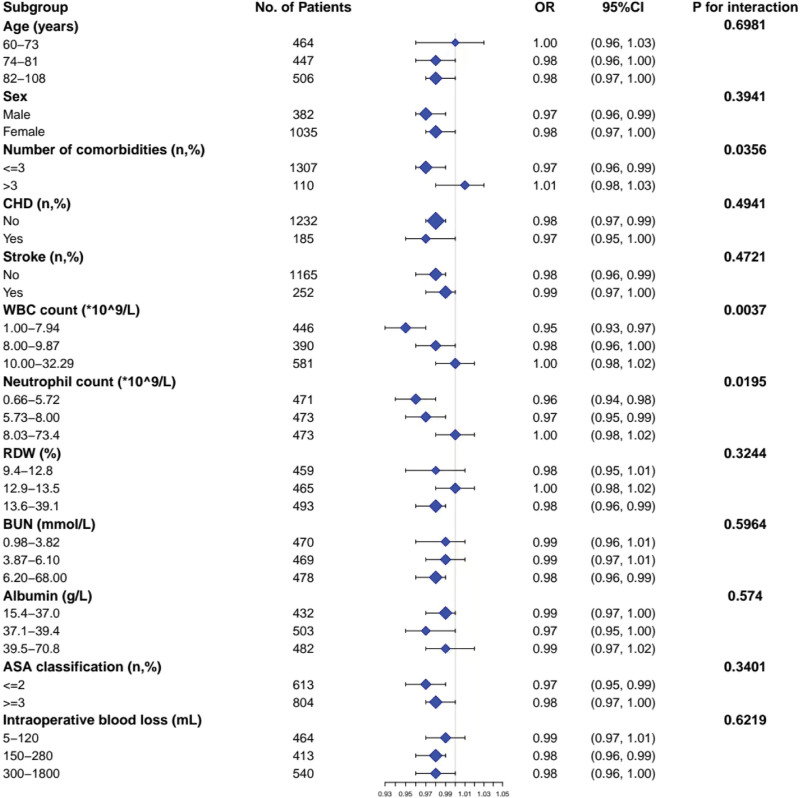
Effect size of hemoglobin on postoperative pneumonia in prespecified and exploratory subgroups. ASA = American Society of Anesthesiologists, BUN = blood urea nitrogen, CHD = coronary heart disease, CI = confidence interval, OR = odds ratio, RDW = red blood cell distribution width, WBC = white blood cell.

As shown in Figure 3, effect modifiers were observed in the relationship between Hb levels and POP. Eligible factors identified as effect modifiers for this relationship included the number of comorbidities (*P* = .0356), WBC count (*P* = .0037), and neutrophil count (*P* = .0195).

## 4. Discussion

The present study investigated the association between Hb levels at admission and POP in elderly patients with hip fractures. Our findings demonstrate the presence of an inflection point in the relationship between Hb levels and POP incidence. Specifically, we observed a 9% reduction in the incidence of POP for every 1.0 g/L increase in Hb on the left side of the inflection point. However, we did not observe a significant correlation between Hb level and POP incidence on the right side of the inflection point.

Previous studies have reported that Hb levels can decrease by more than 20.0 g/L after hip fracture injury.^[16]^ Additionally, the incidence of preoperative anemia in patients undergoing hip and knee replacement ranges from 12.8% to 24.3%.^[8,16]^ Anemia is an independent risk factor for postoperative respiratory complications in patients undergoing surgery.^[17]^ Our study also identified low preoperative Hb level as a risk factor for POP in elderly patients with hip fractures. Previous evidence has suggested that anemia is a significant risk factor for postoperative complications, including POP and mortality.^[18,19]^ This study demonstrated that patients with moderate-to-severe anemia are at an elevated risk of developing POP. Furthermore, a study of individuals infected with coronavirus disease 2019 found a higher incidence of anemia in patients with pneumonia than in those without pneumonia.^[20]^ Therefore, optimizing perioperative treatment and care for patients with anemia is crucial for reducing the incidence of POP after hip fracture surgery.^[21]^

The majority of perioperative blood loss in orthopedic patients is hidden blood loss, which can be 3 to 6 times greater than overt blood loss.^[22–24]^ Moreover, anemia itself can cause and exacerbate gastrointestinal mucosal edema, nausea, dizziness, depression, anorexia, and other problems, leading to a vicious cycle that further aggravates anemia.^[25,26]^ Elderly patients with hip fractures may have weakened anti-infection abilities owing to advanced age, multiple underlying diseases, trauma, surgery, and anesthesia, making them more vulnerable to infectious pathogens and multiple organ dysfunction.^[9,27]^ When a patient has anemia or is on the borderline of having anemia before surgery, perioperative blood loss and poor recovery after surgery further exacerbate anemia. It has been reported that the anemia rate of patients after hip fracture surgery is between 84.6% and 88.5%,^[28]^ and this rate is greater than 80% for patients receiving hip and knee replacements.^[9]^ Therefore, for elderly patients with hip fractures complicated by anemia, it is critical to increase the preoperative Hb level as much as possible to reduce the incidence of POP after hip fracture surgery.^[21]^ These findings underscore the importance of optimizing Hb levels to improve surgical outcomes and to reduce the risk of postoperative complications in this patient population.

Anemia can be corrected by blood transfusion, which has been shown to effectively reduce postoperative complications. Some studies^[29,30]^ have suggested that patients with Hb levels < 80.0 g/L should receive blood transfusions. However, Smeets et al suggested that allogeneic transfusions may increase long-term mortality and cardiovascular events in patients after hip fracture surgery.^[31]^ Our findings indicate that when preoperative Hb levels in elderly patients with hip fractures are less than 83.5 g/L, interventions to improve Hb levels are highly recommended to lower the incidence of POP. It is important for elderly patients with hip fractures to undergo surgical treatment as soon as possible, when their general condition is acceptable. However, it may take a long time for a non-transfusion strategy to improve Hb levels and it is difficult for patients to wait for such a long time for surgical treatment. Transfusion therapy can increase Hb levels within a short period of time to correct anemia. A systematic review suggested that when the Hb level was between 70 and 90 g/L, blood transfusion did not affect the postoperative recovery of patients.^[32]^ According to current clinical practice guidelines, the decision to administer blood transfusions to patients with Hb levels of 70 to 100 g/L should be determined on an individual basis, taking into account factors such as the patient’s age, degree of anemia, cardiopulmonary function, and other relevant factors.^[33]^ Overall, our findings suggest that interventions to improve Hb levels may be critical for reducing the incidence of POP in elderly patients with hip fractures. However, the decision to administer blood transfusions should be made on an individual basis considering the potential risks and benefits of this intervention. Further research is needed to explore optimal strategies for improving Hb levels in this patient population, while minimizing the risk of adverse events. However, there are numerous factors, including several modifiable factors, that contribute to the occurrence of POP in elderly hip fracture patients. A significant factor was the effect of anesthesia was found to be a significant factor. There is still debate regarding the impact of anesthesia on postoperative recovery and complications in patients is still under debate. A randomized controlled trial discovered that spinal anesthesia was not superior to general anesthesia in terms of 60-day survival and return to activity in elderly patients undergoing hip fracture surgery.^[34]^ In contrast, Tian et al identified general anesthesia as an independent risk factor for POP.^[12]^ Another meta-analysis demonstrated that an American Society of Anesthesiologists (ASA) classification of ≥ 3 (OR: 3.48, 95% CI: 1.87–6.47) was a significant independent risk factor for POP in elderly hip fracture patients.^[35]^ However, several studies have found no relationship between different anesthetic techniques and POP in elderly patients undergoing hip fracture surgery.^[36,37]^ This study also did not find any effect of anesthesia on POP. Therefore, future large prospective studies are necessary to explore the effect of the anesthesia modality on POP in elderly patients with hip fractures.

Our study indicates that elderly hip fracture patients with preoperative Hb levels < 83.5 g/L should be considered for blood transfusions to reduce the occurrence of POP. Compared to previous studies, our study included a larger sample size and examined data from elderly patients with hip fractures over a 10-year period. Additionally, we identified an inflection point (83.5 g/L) between preoperative Hb levels and the incidence of POP, which can aid clinicians in clinical decision making regarding blood transfusions.

### 4.1. Limitations

This study had several limitations. First, this was a retrospective study that mainly included elderly patients aged ≥ 60 years. Further prospective studies are required to confirm our findings. Second, this was an observational study and we did not design mechanistic experiments to explain our findings. Third, we only adjusted for measurable confounders but not for unmeasurable confounders. Therefore, future studies with more comprehensive adjustments are required to validate our findings. Aspiration is a significant risk factor of POP in elderly patients with hip fractures. A study^[38]^ reported that the incidence of postoperative aspiration pneumonia in elderly patients with hip fracture was 1.7%, and the incidence of POP was 3.9%. For correctable risk factors, aggressive interventions can help prevent POP and aspiration pneumonia.^[39]^ However, this study did not collect data on whether patients experienced aspiration during the retrospective data collection. Therefore, future studies should include data regarding the effects of aspiration on POP. Similarly, serum ferritin level was not included as a variable in this study, and the effect of ferritin on POP should be examined in future studies. Our study was retrospective and only examined the relationship between preoperative hemoglobin levels and postoperative pneumonia. Therefore, we compare the risk factors between patients with pneumonia and other patients in the attachment (Table S1, Supplemental Digital Content, http://links.lww.com/MD/J330).

## 5. Conclusion

Our study demonstrated that the relationship between preoperative Hb levels and POP was nonlinear in elderly patients with hip fractures. To reduce the occurrence of POP, preoperative intervention measures to improve Hb levels should be implemented in elderly patients with hip fractures when preoperative Hb levels are < 83.5 g/L to reduce the occurrence of POP.

## Acknowledgments

We would like to express our sincere gratitude to Professor Wenlan Liu, Dr Xinglin Chen, and Dr Jun Xia for their valuable guidance and assistance with study design and content. We would also like to acknowledge the financial support provided by Shenzhen Second People’s Hospital for this study. Their contributions were essential to the successful completion of this research project.

## Author contributions

**Data curation:** Daxue Zhang, Yu Zhang, Shiwei Yang.

**Formal analysis:** Ning Zhang.

**Funding acquisition:** Shiwei Yang.

**Methodology:** Shiwei Yang.

**Supervision:** Lixin Sun, Shiwei Yang.

**Writing – original draft:** Daxue Zhang.

**Writing – review & editing:** Shiwei Yang.

## Supplementary Material


