# Supplementary material for: Association of hemoglobin levels at admission with postoperative pneumonia in elderly patients with hip fracture: A retrospective cohort study
Source: Medicine (Baltimore). 2023 Jul 28;102(30):e34270. doi: 10.1097/MD.0000000000034270 (PMC10378990; doi:10.1097/MD.0000000000034270)
Supplement: Supplementary file 1 [file medi-102-e34270-s001.pdf]

Supplemental table: Comparison of postoperative pneumonia between groups

	No POP	POP	Statistics	*P*-value
**Number(n)**	1329	88		
**Age (years)**	77.24 (8.64)	82.47 (8.67)	t=-5.492	<0.001
**Sex (n,%)**			χ^2=4.215	0.040
**Male**	350 (26.34%)	32 (36.36%)		
**Female**	979 (73.66%)	56 (63.64%)		
**BMI (kg/m^2)**	22.06 (3.21)	21.76 (3.85)	t=0.838	0.402
**Classification of fracture (n,%)**			χ^2=0.872	0.350
**Femoral neck fracture**	851 (64.03%)	52 (59.09%)		
**Intertrochanteric fracture**	478 (35.97%)	36 (40.91%)		
**Time from fracture to surgery (hours)**	76.00 (50.00-135.00)	99.50 (59.00-168.50)	Z=-1.597	0.114
**Number of comorbidities (n,%)**			χ^2=8.696	0.003
**≤3**	1233 (92.78%)	74 (84.09%)		
**>3**	96 (7.22%)	14 (15.91%)		
**Smoking status (n,%)**			χ^2=2.431	0.119
**No**	1271 (95.64%)	81 (92.05%)		
**Yes**	58 (4.36%)	7 (7.95%)		
**Hypertension (n,%)**			χ^2=0.623	0.430
**No**	692 (52.07%)	42 (47.73%)		
**Yes**	637 (47.93%)	46 (52.27%)		
**CHD (n,%)**			χ^2=6.022	0.014
**No**	1163 (87.51%)	69 (78.41%)		
**Yes**	166 (12.49%)	19 (21.59%)		
**Stroke (n,%)**			χ^2=14.769	<0.001
**No**	1106 (83.22%)	59 (67.05%)		
**Yes**	223 (16.78%)	29 (32.95%)		
**DM (n,%)**			χ^2=1.069	0.301
**No**	1030 (77.50%)	64 (72.73%)		
**Yes**	299 (22.50%)	24 (27.27%)		
**WBC count (×10^9/L)**	9.17 (2.91)	10.16 (4.51)	t=-2.972	0.003
**Lymphocyte count (×10^9/L)**	1.19 (0.93-1.58)	1.12 (0.77-1.45)	Z=1.149	0.254
**Neutrophil count (×10^9/L)**	7.22 (3.35)	8.31 (4.15)	t=-2.910	0.004
**Monocyte count (×10^9/L)**	0.54 (0.41-0.71)	0.53 (0.40-0.73)	Z=-0.296	0.766
**Platelet count (×10^9/L)**	206.94 (76.04)	203.73 (64.60)	t=0.387	0.699
**RDW (%)**	13.41(1.61)	14.04(1.67)	t=-3.556	<0.001

			Z=-1.615	
**Scr (umol/L)**	64.70 (53.00-80.80)	71.95 (60.15-99.75)		0.110
**BUN (mmol/L)**	5.00 (3.20-6.70)	5.80 (3.92-8.00)	Z=-2.542	0.013
**Albumin (g/L)**	38.18 (4.42)	36.31 (4.62)	t=3.831	<0.001
**Globulin (g/L)**	28.32 (7.21)	30.69 (8.26)	t=-2.975	0.003
**Hemoglobin(g/L)**	117.10 (18.31)	109.44 (23.30)	t=3.733	<0.001
**ASA classification (n,%)**			χ^2=8.431	0.004
**≤2**	588 (44.24%)	25 (28.41%)		
**≥3**	741 (55.76%)	63 (71.59%)		
**Operation method (n,%)**			χ^2=2.041	0.153
**Internal fixation**	556 (41.84%)	30 (34.09%)		
**Hip replacement**	773 (58.16%)	58 (65.91%)		
**Anesthesia method (n,%)**			χ^2=0.000	0.998
**Non general anesthesia**	1012 (76.15%)	67 (76.14%)		
**General anesthesia**	317 (23.85%)	21 (23.86%)		
**Operation duration (min)**	85.25 (35.83)	80.15 (34.71)		0.195
**Intraoperative blood loss (mL)**	200.00 (100.00-300.00)	200.00 (100.00-200.00)	Z=2.964	0.004
**ICU admission (n,%)**			χ^2=82.554	<0.001
**No**	1312 (98.72%)	74 (84.09%)		
**Yes**	17 (1.28%)	14 (15.91%)		
**In-hospital death (n,%)**			χ^2=2.431	0.119
**No**	1326 (99.77%)	87 (98.86%)		
**Yes**	3 (0.23%)	1 (1.14%)		
**Length of hospital stay (days)**	10.00 (8.00-15.00)	16.00 (11.00-20.25)	Z=-4.976	<0.001

Abbreviations: POP, postoperative pneumonia, BMI, body mass index; CHD, coronary heart disease; DM, diabetes mellitus; WBC, white blood cell; RDW, red blood cell distribution width; Scr, serum creatinine; BUN, blood urea nitrogen; ASA, American Society of Anesthesiologists; ICU, intensive care unit
